# Generation of *Phaseolus vulgaris *ESTs and investigation of their regulation upon *Uromyces appendiculatus *infection

**DOI:** 10.1186/1471-2229-9-46

**Published:** 2009-04-27

**Authors:** Sandra Thibivilliers, Trupti Joshi, Kimberly B Campbell, Brian Scheffler, Dong Xu, Bret Cooper, Henry T Nguyen, Gary Stacey

**Affiliations:** 1 National Center for Soybean Biotechnology, Center for Sustainable Energy, Divisions of Plant Sciences and Biochemistry, University of Missouri, Columbia, MO, 65211, USA; 2 Computer Science Department and Christopher S Bond Life Sciences Center, University of Missouri, Columbia, MO, 65211, USA; 3 Soybean Genomics and Improvement Laboratory, USDA-ARS, Beltsville, MD, 20705, USA; 4 MSA Genomics Laboratory, USDA-ARS, Stoneville, MS, 38776, USA

## Abstract

**Background:**

*Phaseolus vulgaris *(common bean) is the second most important legume crop in the world after soybean. Consequently, yield losses due to fungal infection, like *Uromyces appendiculatus *(bean rust), have strong consequences. Several resistant genes were identified that confer resistance to bean rust infection. However, the downstream genes and mechanisms involved in bean resistance to infection are poorly characterized.

**Results:**

A subtractive bean cDNA library composed of 10,581 unisequences was constructed and enriched in sequences regulated by either bean rust race 41, a virulent strain, or race 49, an avirulent strain on cultivar Early Gallatin carrying the resistance gene *Ur-4*. The construction of this library allowed the identification of 6,202 new bean ESTs, significantly adding to the available sequences for this plant. Regulation of selected bean genes in response to bean rust infection was confirmed by qRT-PCR. Plant gene expression was similar for both race 41 and 49 during the first 48 hours of the infection process but varied significantly at the later time points (72–96 hours after inoculation) mainly due to the presence of the *Avr4 *gene in the race 49 leading to a hypersensitive response in the bean plants. A biphasic pattern of gene expression was observed for several genes regulated in response to fungal infection.

**Conclusion:**

The enrichment of the public database with over 6,000 bean ESTs significantly adds to the genomic resources available for this important crop plant. The analysis of these genes in response to bean rust infection provides a foundation for further studies of the mechanism of fungal disease resistance. The expression pattern of 90 bean genes upon rust infection shares several features with other legumes infected by biotrophic fungi. This finding suggests that the *P. vulgaris*-*U. appendiculatus *pathosystem could serve as a model to explore legume-rust interaction.

## Background

Common bean, *Phaseolus vulgaris*, represents a great source of nutrition for millions of people and is the second most important legume crop, after soybean. It is the target of multiple pests and diseases causing substantial losses. For example, on susceptible bean cultivars, bean rust, caused by *Uromyces appendiculatus*, may cause yield reduction from 18 to 100% with favorable environmental conditions, such as high moisture and temperature between 17 and 27°C [1]. Among the 5 different stages of the bean rust life cycle, basidia, pycnia, aecia, uredinia, and telia, the most devastating on bean is the uredinial stage. The latent period between the germination of an urediniospore and the formation of a sporulating pustule can be as short as 7 days. Signs of infection by *Uromyces appendiculatus *include the presence of uredinia or spore-producing pustules on the surface of the leaf. The identification of fungal proteins from quiescent and germinating uredospores enhanced the understanding of the infection process of this fungus [2,3].

Based upon mapping and quantitative trait loci (QTL) analysis, several genes involved in *Colletotrichum lindemuthianum *(Co; anthracnose)resistance and other resistance genes for bean common mosaic virus (BCMV), bean golden yellow mosaic virus (BGYMV), common bacterial blight, and bean rust are clustered [2,3]. The large number of resistance (R) genes for bean rust may correlate with the high pathogen population diversity; with 90 different races identified [4]. The locus *Ur-3 *confers resistance to 44 out of the 89 *U. appendiculatus *races present in the USA [5,6]. Besides the *Ur-3 *locus, a number of other R genes were identified in bean; such as locus *Ur-4 *for race 49, locus *Ur-11 *epistatic to *Ur-4 *for race 67 or locus *Ur-13 *mapped to the linkage group B8 [7,8]. To date, no large scale transcriptomic analysis of bean rust infection has been performed to better understand the mechanism of resistance. All of these *Ur *genes are effective against one specific rust strain, following the gene-for-gene resistance theory. Consequently, gene pyramiding was used to produce cultivars carrying multiple resistance genes [9]. Unfortunately, such resistance may prove to be effective in the field for only a short time due to the adaptation of the fungus to overcome plant defenses [10]. Consequently, unraveling and understanding the mechanisms downstream of these R genes is a key research goal to circumvent the adaptation of the fungus to plant resistance.

We investigated the *Phaseolus vulgaris-Uromyces appendiculatus *pathosystem at a transcriptional level for a better understanding of the plant response to fungal infection. In this study, we developed a subtractive suppressive hybridization (SSH) library made from the common bean cultivar Early Gallatin that exhibits susceptibility to *U. appendiculatus *race 41(virulent strain) but resistance to *U. appendiculatus *race 49 (avirulent strain). The resistance to *U. appendiculatus *is conferred by the presence of the *Ur-4 *gene in this cultivar that leads to a hypersensitive response (HR) in presence of the pathogen race 49 [11]. This cDNA bean library was enriched in expressed sequence tags (ESTs) that are potentially up-regulated by the compatible and incompatible interactions. More than 20,000 clones from the SSH library were sequenced and assembled into contigs. A total of 10,221 *P. vulgaris *sequences and 360 *U. appendiculatus *sequences were added to the NCBI database, significantly increasing the number of ESTs available for common bean. The regulation of 90 genes was confirmed by quantitative real time polymerase chain reaction (qRT-PCR) revealing 3 main expression patterns and highlighting gene regulation that occurs downstream of R protein activation.

## Results and discussion

### Identification of unisequences from tissues infected with virulent or avirulent bean rust

Common bean is a diploid (n = 11) with a small genome size estimated at 450 to 650 Mb [12]. So far, the total number of common bean ESTs available is 83,448 (verified on March, 2009). This number was significantly less before the publication of [13] who added ESTs from root nodules, phosphorus deficient roots, developing pods, and leaves, and from leaves and shoots with and without *C. lindemuthianum *inoculation [14]. (The current number also includes the 10, 221 ESTs added in this study.). The lack of sufficient *P. vulgaris *sequences precludes the construction of a useful DNA microarray for this plant. Consequently, in order to study the response of bean to *U. appendiculatus *infection, we created a SSH library and sequenced 20,736 clones from 3' and 5' ends. From 41,472 sequences, 8.5% were discarded due to the absence of a cloned sequence or low sequence quality.

**Figure 1:**
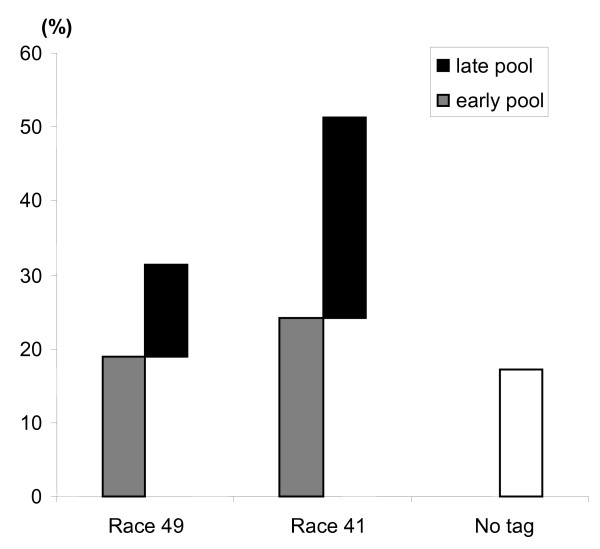
**Distribution of the sequences according to their tag**. The EST sequences are representing in the grey or black columns depending on whether they came from tissues harvested early (6 to 24 HAI) or late (48 to 120 HAI). X-axis represents the fungal race with which the leaves were infected prior to cDNA isolation. Y-axis represents the percentage of sequences in each category *versus *the total number of sequences of the library.

During cDNA generation, sequence tags were incorporated prior to pooling cDNAs from different conditions (see Material and Methods for details). The tags identify the treatment and time points used in generating the original mRNA. The distribution of these tags among the library is presented in the Figure 1. Approximately 17% of the sequences lacked a tag after sequencing, while 31% of the sequences had a "race 49" tag and more than 51% had a "race 41" tag. It is important to note that the majority of the ESTs coming from the fungus were tagged "late41", consistent with an effective colonization of the leaf by the virulent fungus (race 41). The lack of tag identification may come from inefficient incorporation of the tag during the library construction or the presence of non-identified nucleotide in the tag sequence making it indiscernible. The various cDNAs in the library could be resolved back to their source tissue by the presence of unique sequence tags. For example, 51% percent of the EST sequences were derived from bean tissue infected with race 41 since they had the "race 41" tag. This likely reflects the compatible interaction between the race 41 and its host allowing greater fungal penetration. Biotrophic fungi are known to reprogram the host plant cell to support their growth [15] and plant ESTs tagged with race 41 could be involved in this process.

Contig assembly and removal of redundant sequences was performed on 38,592 sequences using TIGR Gene Indices Clustering Tools (TGICL) and CAP3 software. Two thousand seven hundred twenty one sequences showed no similarity with other sequences and were categorized as singletons. These sequences had an average length of 670 bp. Seven thousand eight hundred sixty contigs were assembled from the remaining 35,163 sequences. The average contig length was estimated to be 1 kb. An average contig contains 4.5 sequences (min:2, max:496). Ultimately, 10,581 unisequences were identified and represent genes that are potentially differentially up-regulated during bean infection by virulent or avirulent pathogen isolates.

Among these 10,581 unisequences, 10,221 were annotated as bean genes and 360 were annotated as fungal genes based on best Blast hits to the database These 360 fungal unisequences included 62 singletons and 298 contigs (Table 1).

**Table 1:** Distribution of the ESTs according to the genus giving the best hit (E-value ≤ e^-20^)

	Unisequence	singleton	contig = 2	contig>2
vector	13	2	7	4
No hit	118	49	66	3
Low hit (>e^-20^)	1279	514	663	102
Fungi	360	62	210	88
Barley	1	1	0	0
Ice plant	1	0	1	0
Petunia	1	1	0	0
*S. pombe*	1	0	1	0
Sugarcane	1	1	0	0
Sunflower	1	0	1	0
Beet	2	1	0	1
*N. benthamiana*	2	1	1	0
Oilseed rape	2	0	0	2
Tobacco	2	1	1	0
Pinus	3	3	0	0
Potato	3	2	1	0
Letuce	4	2	2	0
Wheat	5	2	0	3
Aquilegia	6	3	2	1
Rice	6	5	0	1
Tomato	7	0	5	2
*A. thaliana*	9	4	3	2
Poplar	15	6	5	4
Grape	18	6	9	3
Cotton	23	8	13	2
Lotus	145	51	52	42
*M. truncatula*	500	190	184	126
Common bean	3473	622	1249	1602
Soybean	4580	1184	1844	1552
Total	10581	2721	4320	3540

"Unisequence", "singleton", "contig = 2", and "contig>2" columns represent the number of total unisequences, those found once in the SSH library, the contigs made up of only 2 sequences and the contigs composed of more than 2 sequences, respectively, having a hit with an organism listed in column one.

### Functional annotation

**Figure 2:**
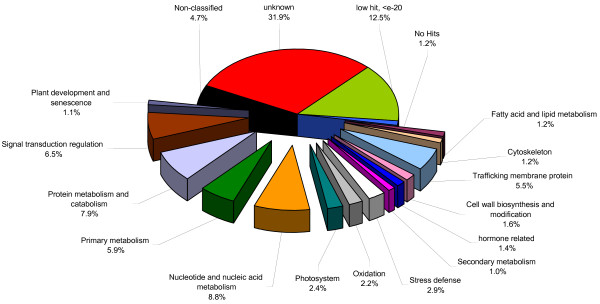
**Functional distribution of the 7,851 contigs and 2,719 singletons based on homology (E-values ≤ e^-20^)**. The sequences can be grouped in 2 main categories, "no annotation" (50.3%), "annotated EST" (49.7%), and subdivided into 18 subcategories as shown.

Sequence analysis revealed that 8,806 ESTs had significant similarity with sequences in public databases such as DFCI or NCBI (using BlastN with an E-value ≤ e-20). Forty-three percent of annotations were based on similarities to sequences in soybean databases and 32.8% were derived from comparisons with common bean (Table 1, see additional file 1: excel file of the 10,581 unisequences). These unisequences were grouped into 18 different functional categories (Figure 2). The most abundant category contained the unknown (31.9%), non-classified (4.7%), and low or no hit (13.7%) groups and represents 50.3% of the entire library. The remaining 49.7% of the sequences were grouped into 14 categories, such as, protein metabolism and catabolism (7.9%), nucleotide and nucleic acid metabolism (8.8%), or stress defense response (2.9%). Taken together, signal transduction regulation and nucleotide and nucleic acid metabolism represent 15.3% of the library. Tian *et al. *(2007) also found that 14% of their EST library, made from phosphorus starved bean plants, fell into these two categories [16]. Similar observations were made on soybean in response to stresses such as drought, phosphorus starvation or nematode infection [17]. It would seem that under various biotic and abiotic stresses, plants activate several common pathways that alter the expression profile of genes, which allow the plant to response to the specific environmental condition.

The SSH library was normalized to reduce the redundancy of the most highly expressed genes. However, some genes are very highly expressed and thus remain overrepresented in the normalized library. As expected, the largest contigs (i.e., composed of the most sequences) are involved in basic metabolism processes. The primary metabolism category comprises the largest component (5.9%) of the library based upon the number of unisequences and the proportion of contigs composed of a high number of sequences. For example, CL1contig48 is composed of 496 aligned sequences and not surprisingly, represents ribulose-1,5-bisphosphate carboxylase/oxygenase activase (RuBisco activase). Three of the other 15 largest contigs are also found in the primary metabolism category (Table 2, see additional file 1: excel file of the 10,581 unisequences).

**Table 2:** List of the 15 most abundant bean contigs with the highest number of sequences

Total No. of ESTs	Contig annotation	Category	genus	gene annotation*
496	CL1Contig48	primary metabolism	common bean	Ribulose bisphosphate carboxylase/oxygenase activase, chloroplast precursor (RubisCO activase)
358	CL1Contig437	Amino acid and protein metabolism	common bean	large subunit 26S ribosomal RNA gene
318	CL1Contig308	Amino acid and protein metabolism	Lotus	large subunit 26S ribosomal RNA gene
220	CL1Contig62	energy	common bean	Chlorophyll a/b-binding protein
175	CL1Contig88	primary metabolism	common bean	Glyceraldehyde-3-phosphate dehydrogenase A, chloroplast precursor (NADP-dependent glyceraldehydephosphate dehydrogenase subunit A)
164	CL1Contig147	energy	common bean	LHCII type II chlorophyll a/b-binding protein
151	CL1Contig39	Amino acid and protein metabolism	common bean	Cysteine protease
143	CL2Contig1	energy	common bean	Oxygen-evolving enhancer protein 1, chloroplast precursor (33 kDa subunit of oxygen evolving system of photosystem II)
121	CL1Contig159	Amino acid and protein metabolism	common bean	Human ribosomal DNA complete repeating unit
106	CL1Contig89	oxidation	common bean	glycolate oxidase. (Lens culinaris)
101	CL1Contig123	primary metabolism	common bean	Glyceraldehyde 3-phosphate dehydrogenase
98	CL1Contig301	primary metabolism	common bean	Plastidic aldolase
92	CL2Contig2	energy	common bean	Oxygen-evolving enhancer protein 1, chloroplast precursor (33 kDa subunit of oxygen evolving system of photosystem II)
88	CL3Contig1	Amino acid and protein metabolism	common bean	T6D22.2
80	CL1Contig218	oxidation	Soybean	F1E22.18

* Based on the BlastX hits (E value > e -20) with ESTs deposited in the dbEST at NCBI

This library was constructed to reveal the plant and fungal genes up-regulated during the rust infection process. Contigs correlated with stress response pathways also have a high number of sequences such as the contig CL1contig105 with 72 sequences encoding an 1-aminocyclopropane-1-carboxylic acid oxidase (ACC oxidase), the contig CL10contig1 with 55 sequences encoding for a glucan endo-1,3-beta-glucosidase, and the contig CL22contig1 with 37 sequences and encoding an endochitinase.

To determine the proportion of new *P. vulgaris *unigenes among the library, the sequences were compared with the *P. vulgaris *ESTs present in the NCBI database. By this analysis, 6,202 sequences, out of the 10,221 bean ESTs, can be considered as new *P. vulgaris *unigenes with the remaining 4,019 sequences matching known sequences with more than 98% identity over more than 100 bp. The ESTs present in the NCBI database originate from common bean cultivars such as Bat93, Negro Jamapa 81 or G19883, facilitating the identification of putative single nucleotide polymorphisms (SNPs) between these public sequences and the ESTs derived from this study using cultivar Early Gallatin. Of the 4,019 matching sequences, 791 sequences present a perfect match, 762 sequences have 1 mismatch or indel, 658 have 2 mismatches and/or indels, and 1,807 have more than 3 mismatches and/or indels. An average of 1 SNP/indel is putatively identified every 335 bp. However, we were not able to further confirm these SNP/indels due to the lack of the sequence trace files for the bean ESTs present in the NCBI database. This SNP frequency is very similar to that reported previously by Ramirez *et al.*, (2005) who found 1 SNP every 387 bp. Our estimation is based on the comparison of cv Early Gallatin with 3 other cultivars (Bat93, Negro Jamapa 81, and G19883). When this comparison is made between only 2 different cultivars (Early Gallatin and G19883) the SNP frequency in the coding sequences decreases to 1 SNP every 570 bp. For comparison, the SNP frequency in the soybean coding sequence was estimated at 1 SNP/490 bp in exons and 1 SNP/375 bp in introns [18]. The genes identified by EST sequencing represent candidates involved in the plant host's ability to withstand rust infection. Therefore, genetic mapping of these gene candidates is a means to correlate their position with known QTL involved in disease resistance.

The 360 fungal sequences represent 3.4% of the library. Two studies in rice showed that the harvesting time (i.e., fungal biomass in the infected leaf is low at the earliest time points) and the stringency of selection (i.e., choice of the appropriate E-value for the blast) are very important to accurately sample the abundance of fungal EST in infected leaf tissue [19,20]. In this study, the selected E-value was e-20, greatly reducing the risk of false positive clones. Tissue was sampled after 5 days of infection allowing the multiplication of the fungi in the leaf tissue. At 5DAI, the haustoria are already mature and are probably redirecting the nutrient up-taken from the plant based on their genes expression pattern [21].

These genes were mainly annotated predominantly by comparison to ESTs from germinating uredospores of *U. appendiculatus *[22,23] (Table 3, see additional file 1: excel file of the 10,581 unisequences). Two hundred seventy two fungal sequences, representing 74.4% of the total, were considered identical to ESTs already present in the NCBI database while 88 sequences are new and unique as identified by less than 98% identity over at least 100 bp. Interestingly, among the 88 fungal ESTs that showed no similarity with ESTs from *U. appendiculatus *germinating uredospores, 19 showed similarity with *Uromyces viciae *haustorium-specific cDNAs and may be specific to successful infections. These remaining sequences represent candidates for fungal genes more directly involved in the infection mechanism. The library was made from tissues infected with a virulent and avirulent rust strain to allow for the identification of genes involved in both pathogen-host compatibility and resistance. Beside, the high similarity of these 19 sequences with haustoria-specific ESTs makes them likely candidates to encode potential effectors or avirulence proteins.

**Table 3:** List of the 15 most common fungal rust contigs containing the highest number of sequences

Total ESTs in contig	Contig annotation	genus	gene annotation *
18	CL1Contig467	*Phakopsora pachyrhizi*	cDNA from germinating urediniospores SSH-library similar to beta-galactosidase
14	CL229Contig1	*Uromyces viciae*	haustorium-specific cDNA similar to mitochondrial substrate carrier
14	CL201Contig1	*Uromyces appendiculatus*	cDNA from hyphae from gernimating uredospore similar to translation elongation factor
14	CL1Contig310	*Uromyces appendiculatus*	cDNA from hyphae from gernimating uredospore similar to von Willebrand factor
12	CL1Contig460	*Phakopsora pachyrhizi*	cDNA from germinating urediniospores SSH-library similar to von Willebrand factor
10	CL124Contig1	*Uromyces appendiculatus*	cDNA from hyphae from gernimating uredospore similar to unknown
10	CL116Contig1	*Uromyces appendiculatus*	cDNA from hyphae from gernimating uredospore similar to unnknown
10	CL492Contig1	*Uromyces appendiculatus*	cDNA from hyphae from gernimating uredospore similar to unknown
8	CL633Contig1	*Uromyces viciae*	haustorium-specific cDNA similar to nucleotide excision repair protein yeast rad23
8	CL662Contig1	*Uromyces viciae*	haustorium-specific cDNA similar to 6-phosphogluconate dehydrogenase
8	CL116Contig2	*Uromyces appendiculatus*	cDNA from hyphae from gernimating uredospore similar to unknown
8	CL766Contig1	*Puccinia graminis f. sp. tritici*	SSH-library of Puccinia graminis infected wheat leaves similar to 60s ribosomal protein L5 gene
8	CL772Contig1	*Uromyces viciae*	haustorium-specific cDNA similar to voltage-dependent ion-selective channel
8	CL582Contig1	*Puccinia graminis f. sp. tritici*	SSH-library of Puccinia graminis infected wheat leaves similar to glutathione S-transferase
8	CL787Contig1	*Uromyces appendiculatus*	cDNA from hyphae from gernimating uredospore similar to cysteine-rich secretory protein (CRISP/SCP/TPX1)

* Based on the BlastX hits (E value > e -20) with fungal ESTs

The largest contig has sequence similarity to a putative beta-galactosidase (an enzyme involved in the degradation of the cell-wall) based on a match to a cDNA from germinating *P. pachyrhizi *uredospores.

### Comparative analysis with the *Phaseolus vulgaris *BAC-end sequences

Recently, the University of Purdue released the first bean FingerPrinted Contigs (FPC) physical map that contains 7,567 contigs or singletons and is anchored with 240 genetic markers . The Bacterial artificial chromosome-ends (BAC) of the clones were sequenced and provide a powerful tool for integrated genomic and genetic analysis. This recent release of the *Phaseolus *physical map  allowed linkage of some of the 10,221 unisequences to the physical map by comparison to BAC-end sequences. The number of ESTs matching a BAC-end sequence was assessed using the minimal criteria of 98% sequence identity spanning 100 bp. As a result, 1,704 unisequences, more than 15% of the total library, could be linked to the physical map (see additional file 2: excel file of the 1,704 unisequences having a hit with a BAC-end sequence). Fourteen of these sequences located to physical contig 1 that is composed of 999 BACs. The genetic mapping of these ESTs might be facilitated by the presence of genetic markers anchoring BACs within the various contigs.

This physical map was made from common bean cultivar G19833. The number of putative SNP between the BAC-end sequences and the ESTs (common bean cultivar Early gallatin) was identified among the 1,704 matching ESTs. Six hundred seventy ESTs showed a perfect match with a BAC-end sequence, 414 ESTs exhibited only 1 mismatch, 258 ESTs contained 2 mismatches and 362 ESTs had 3 or more mismatches. This represents an average of 1 SNP/indel every 570 bp.

### Identification of bean reference genes

Among the 10,221 unisequences, we sought to confirm the expression of 90 ESTs using qRT-PCR. To normalize gene expression based on qRT-PCR, the identification of constitutively expressed bean reference genes is required. The use of house keeping genes as reference genes for gene expression normalization can induce some error in the analysis of the data without confirmation of their constitutive expression especially when using qRT-PCR [24,25]. Consequently, three bean genes, *TC197*, *TC127*, and *TC185 *(encoding a guanine nucleotide-binding protein beta subunit-like protein, ubiquitin, and tubulin beta chain respectively) were selected based on their housekeeping function and/or their presence in different bean cDNA libraries [13,14]. Additionally, homologs of soybean genes *cons6*, *cons7*, and *cons15 *(encoding for a F-box protein family, a metalloprotease, and a peptidase S16 respectively), were chosen since they were recently shown to be expressed constitutively in soybean [26].

**Figure 3:**
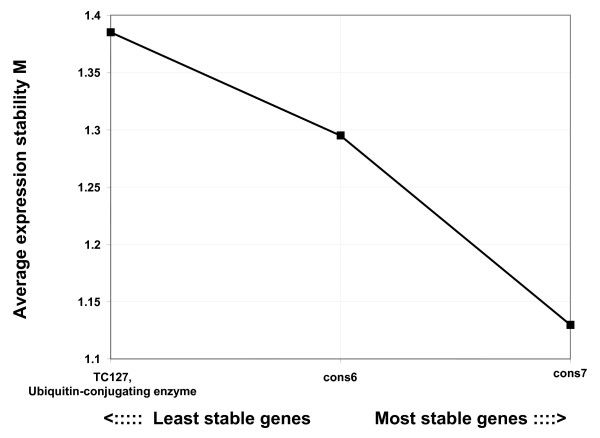
**Ranking of bean genes based on their expression stability measured by qRT-PCR**. The expression levels of three putative constitutive genes (*TC127*, *cons6*, and *cons7*) was measured during infection by both fungal race 41 and 49 in order to identify the best reference gene for qRT-PCR normalization. Genes with the most stable expression during the conditions tested are on the right of the diagram, the less stably expressed being on the left. Figure generated by geNorm software.

Preliminary analysis of these putative constitutive genes by qRT-PCR performed on leaf, stem, and pod cDNA led to the elimination of *TC197*, *cons15 *and *TC185 *due to the variability of their expression levels (data not shown). The stability of the expression level of the 3 remaining genes, *TC127*, *cons6 *and *cons7 *was evaluated by qRT-PCR on cDNAs from bean uninfected or infected with bean rust race 41 or 49 at 6, 12, 24, 48, 72, and 96 hours after inoculation (HAI). After analysis of their expression stability using geNorm software [27], *cons7 *was the most stably expressed in our experimental conditions (Figure 3). For this reason, *cons7 *was selected for normalization of the expression data. It is interesting to note that *cons7 *was also among the most stably expressed constitutive genes in soybean [26] and, therefore, could be a candidate to use for expression normalization in other legumes.

### Transcriptional analysis of selected ESTs during the bean rust infection process

**Figure 4:**
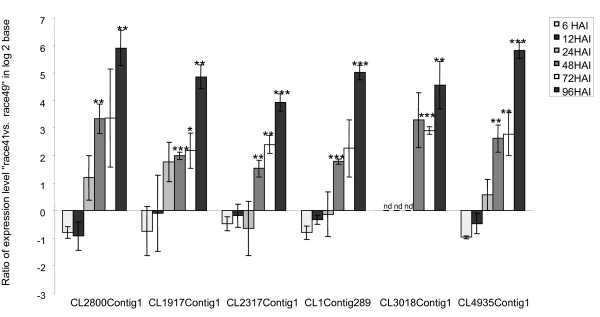
**Transcriptional expression of selected fungal genes during the infection process**. Expression ratio of selected *U. appendiculatus *genes during the first 96 hours of the infection with bean rust race 49 or 41. qRT-PCR was performed on three independent biological replicates using *Cons7 *as a reference for normalization. Six ESTs, CL2800Contig1 (heat shock protein 90), CL1917Contig1 (proteasome subunit alpha), CL2317Contig1 (Glutamine synthetase), CL1Contig289 (Asparaginyl-tRNA synthetase), CL3018Contig1 (planta-induced rust protein 1), and CL4935Contig1 (unknown), were found strongly up-regulated in tissues infected with the fungal race 41 in comparison to tissues infected with the fungal race 49. The tag identification for these ESTs is "late race 41" indicating that they came originally from tissue infected with race 41. *: data significant with 0.05 < p-value ≤ 0.1. **: data significant with 0.01 < p-value ≤ 0.05. ***: data significant with p-value ≤ 0.01. nd: not determined.

In order to compare expression of genes responding to *U. appendiculatus *race 41 to those responding to race 49, during bean infection and colonization, the expression level of six, selected fungal genes was analyzed using qRT-PCR (Figure 4). During the first 24 hours of the infection, the six genes were expressed at comparable levels. However, by 48 HAI, the expression of all six genes was significantly higher in tissues infected with the virulent race 41 isolate. This result likely reflects the nature of the compatible, virulent interaction as compared to inhibition of race 49 infection by the host defenses. Consistent with this, all six genes used in this analysis came from the ESTs possessing the tag of the "late41" library. The EST CL3018Contig1, encoding for a plant-induced rust protein 1, exhibits significant similarity with NMT1 (no messenger in thiamine), which is involved in the biosynthesis of the pyrimidine moiety of thiamine (vitamin B1). This gene was strongly expressed only in tissue infected with the virulent fungus race 41. Similar observations were made previously using bean plants infected with *Uromyces fabae *[28]. These data also suggest that the haustoria may not only be the site of nutrient uptake from the plant [29] but also the site of metabolite biosynthesis with specific haustorial genes involved in vitamin biosynthesis [e.g., NMT1][28].

Ninety bean unisequences were selected (based on their putative function and tag) and their regulation was confirmed by qRT-PCR using RNA obtained from three independent biological replicates. Unisequences coming from the ESTs in the "race 49" tagged libraries were desirable due to their potential involvement in a resistance pathway. The regulation of these genes was evaluated by qRT-PCR using RNA from uninoculated leaf tissues or those inoculated with either *U. appendiculatus *uredospores of race 41 or race 49 isolates at the time points 0, 6, 12, 24, 48, 72, or 96 HAI. The data obtained were used to compare the ratio of gene expression in tissues infected with race 41 or race 49 to that in uninoculated bean leaves. The data also allowed a direct comparison of gene expression induced by either race 41 or race 49. The first two comparisons highlight regulation in the infected plants by the rust fungi, while the third comparison highlights gene expression differences between the two types of infection, resistant and susceptible. The 90 genes showed significant expression differences in at least one of the 3 comparisons (p-value < 0.05, cut-off < -1 or > 1 or p-value < 0.1, cut-off < -0.58 or > 0.58 in log base 2).

**Figure 5:**
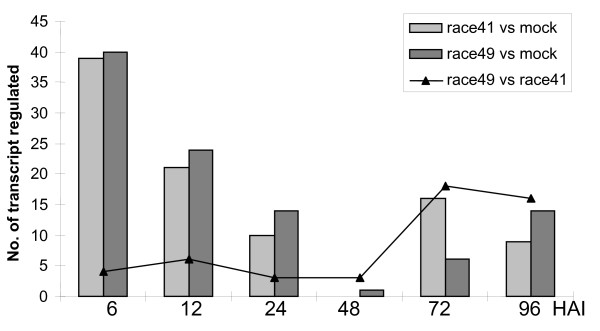
**Temporal expression pattern of the 90 regulated transcripts during the infection process**. Columns light and dark gray represent the number of transcripts regulated in bean infected by race 41 or 49, respectively, in comparison to uninoculated bean plants. The black triangles represent the number of transcripts differentially regulated in the bean infected by race 49 in comparison to the plants infected by race 41.

The transcriptional response was profiled in relation to the time after inoculation (Figure 5). For example, 39 and 41 genes showed differential regulation within 6 and 12 HAI, respectively, in tissue inoculated with race 41. At these same time points 40 and 24 genes, respectively, were differentially regulated in tissues infected with race 49. At the latest time points, 72 and 96 HAI, 16 and 19 genes, respectively, for race 41 and 6 and 14 genes, respectively, for race 49 were differentially regulated. It is interesting to note that the regulation occurring at the early time points appeared to be independent of the fungal race used for inoculation. At the early time points (i.e., first 48 hours), only 16 genes (36% of those tested) showed a difference in expression in tissues inoculated with the two fungal races. However, at the later time points, this number increased to 34 genes with 18 (36%) and 16 (32%) at 72 and 96 HAI, respectively. These results suggested that during the beginning of the infection most of the bean gene regulation is independent of the fungal race, but differences due to fungal race occur as the infection progresses. It is possible that fungal-Pathogen Associated Molecular Pattern (PAMP) elicitors (e.g., chitin) induce the same response from the plant at the beginning of the infection. Subsequently, the Avr4 protein in the race 49 is recognized after a couple of days leading to the induction of defense-related genes. However, in bean infected by race 41, no plant defense is activated and gene expression may reflect the reprogramming of the plant host by the fungus especially at the haustorial site.

A key finding of the van de Mortel *et al*. (2007) study on *Glycine max *– *Phakopsora pachyrhizi *was that most genes were regulated early during infection (the first 24 hours) and at the latest time points tested (72–120 hours). However, at the intermediate time points (24 to 72 hours), few genes were regulated; this phenomenon was called a "dip" by van de Mortel *et al. *(2007). This same expression profile was also observed in bean upon rust infection by both races. At 24 and 48 HAI few genes were regulated in comparison to 6–12–72 and 96 HAI. In common bean, those genes regulated at 6 HAI were very different from those expressed at 72–96 HAI (Figure 5). Therefore, the dip pattern of gene expression upon rust infection appears to occur in both bean and soybean. Furthermore, this biphasic regulation seems to be shared not only by rust fungi but by other biotrophic fungi. For example barley infected by *Blumeria graminis *(causal agent of the powdery mildew), also showed a biphasic gene response, the first set of genes responded in the first 24 hours of the infection in the epidermis whereas the second set responded after 72–96 hours of infection in the mesophyll cells [30]. In contrast, soybean plants infected with *Phytophthora sojae*, a hemibiotrophic oomycete, did not show a biphasic pattern of gene response [31]. Based on these examples, this biphasic pattern might be specific to the biotrophic rust fungi. Further comparisons need to be made to establish the specificity of this "dip" pattern of gene expression in response to biotrophic fungal infection.

**Figure 6:**
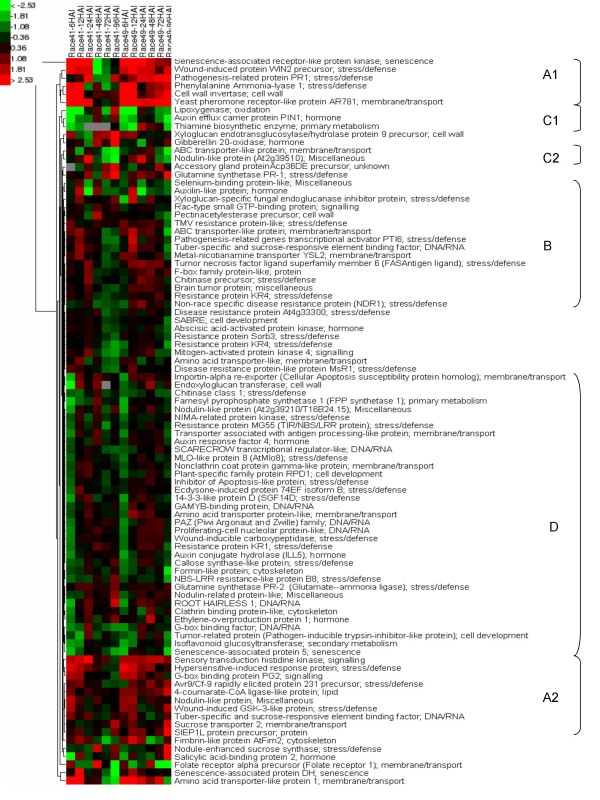
**Transcription profile of the bean genes during rust infection**. Hierarchical clustering of the expression ratio of the ESTs differentially expressed in the inoculated bean by race 41 or 49 in comparison to uninoculated bean tissue. qRT-PCR was performed on 3 independent biological replicates. Analyses were performed on the data set in a Log 2 form based on Euclidean distance.

A more detailed analysis was performed on the expression ratio of transcripts in bean leaves inoculated with the fungus race 41 or race 49 versus uninoculated bean leaves. These analyses are presented in a hierarchical cluster based on Euclidian distance (Figure 6, see additional file 3: excel file of the ratio of the expression level of the 90 regulated genes for all conditions). This cluster can be divided into five main groups. The first, group A (A1 and A2), is composed of 17 genes up-regulated by both fungal races in the first 24 hours of the infection but enhanced expression is subsequently maintained only in the plants infected by race 49 at the later time points (up to 96 HAI). Genes in this group include those annotated as plant defense (35% of this group) containing PR1, wound induced protein 2 (WIN2) genes, cell-wall related (i.e., a cell-wall invertase gene), and signal transduction regulation category with a G-box binding protein PG2 or sensory transduction histidine kinase genes. These genes are likely involved in the defense pathways induced by a fungal-PAMP since they have the same expression pattern during the first 24 hours of infection independent of the fungal race used. For example, the wound-induced protein *WIN2 *protein has anti-fungal activity [32] and possesses a domain that can bind a well known PAMP, chitin [33]. The formation of haustoria by the fungus in the plant can occur within hours of infection [34]. After successful colonization of the bean cell, rust race 41 likely secretes effector proteins that can suppress the plant defense pathway induced by PAMPs. The initial induction of genes such as WIN2 by race 41 and their subsequent reduction in expression may be associated with this suppression of defense by the virulent pathogen only. The second group, group B, is composed of 16 genes that were induced at the beginning of the infection but were slightly down-regulated at the later time points independent of the fungal race. This group is rich in genes categorized as plant defense representing 56% of this group. The third group, group C (C1 and C2), is composed of 5 genes that appeared to be repressed by inoculation. In group C1, the genes were repressed during the first 12 hours by both races but this repression was only maintained at later time points (i.e., 72 and 96 HAI) in tissues infected with race 49. In contrast to group C2, the apparent repression of genes occurred only after 72 HAI with both races. The fourth group, group D, consists of 35 genes that were repressed in the first 12 hours by both races and subsequently expressed at levels comparable to the uninfected tissue. The genes repressed specifically at the early time points could also be involved in the basal defense response. This pool is composed of ESTs known to be involved in plant defense pathways [e.g., chitinase class 1 [35], an auxin response factor 4 and an auxin conjugate hydrolase [36], and a MLO-like protein 8 [37]]. Finally, the fifth group represents 18 genes that gave no discernable pattern of expression.

These 90 representative genes mainly identified genes involved in the early responses of the bean under rust infection (i.e., first 96 HAI). These genes share different expression patterns but are likely involved in the basal defense response, which is induced by PAMPs. These genes were induced by both races at the early time points but their regulation was often maintained only in plants infected by the fungus race 49. This may be due to the inability of this avirulent pathogen to suppress the plant defense system. The same observation was made also by Lee *et al*. (2008) at the protein level. Lee *et al. *(2008) proposed a new model for plant disease resistance where R-gene mediated resistance is integrated into the basal immunity system of the plant and functions primarily to restore the innate immunity response that is actively suppressed by virulent pathogens[38]. Similar patterns of expression, independent of the pathogen virulence, were observed in Arabidopsis [39] and barley [40]. Another category of genes (i.e. cell-wall invertase or amino acid transporter-like protein 1) involved in the plant defense system are likely involved in the HR and were regulated only at the later time points in plants infected by the race 49 fungus. The expression of these genes may be the result of recognition of AvrUR-4 by the Ur-4 resistance protein and lead to the presence of HR ten days after infection with this isolates.

## Conclusion

In summary, we identified 10,581 *P. vulgaris *unisequences and confirmed the regulation of 90 plant genes by rust infection in common bean. These data have added significantly to the genomic resources available for common bean, while also providing insight into how this plant responds to fungal infection. As part of this study, we identified constitutively expressed bean genes that can be used for normalization in gene expression studies. The data also suggest that a biphasic gene expression pattern may be a common feature in plants infected by biotrophic fungi.

## Methods

### Plant material

Bean tissues were produced at the USDA-ARS facility (Beltsville, MD). *P. vulgaris *cv. Early Gallatin plants were inoculated with either *U. appendiculatus *race 41 (virulent strain) or race 49 (avirulent strain) uredospores. The primary leaves of 10 day old plants were inoculated on the top and bottom. Spores (2 × 10^5^spores/ml) were mixed in water and then sprayed on leaves with an aerosol canister. The plants were placed in a dew chamber in the dark at 20°C for 12 hours and then moved to a growth room (24°C, 90% relative humidity) with supplemental fluorescent lighting (12 hours light/12 hours light). Leaves were harvested 0, 6, 12, 24, 48, 72, 96, and 120 HAI in 3 independent experiments. The presence of pustules or HR lesions when inoculated with *U. appendiculatus *race 41 or 49 isolates was observed 10 days after inoculation. Bean leaf, stem, and pod tissues used for the identification of the putative constitutive genes were harvested on 3 month old plants grown in a greenhouse.

### SSH library construction

The normalized SSH library was generated at the Roy J. Carver Biotechnology Center (Urbana, IL). The library is composed of more than 20,000 ESTs and was prepared as described by Bonaldo *et al. *(1996) following the 4^th ^method [41]. The cDNA from bean infected with *U. appendiculatus *race 41 or 49 was pooled and tagged as follows, early41/49 and late41/49 for cDNA from bean tissues infected for 6–12–15–24 or 48–72–96–120 hours, respectively, by either race 41 or race 49. The enrichment in cDNA regulated by the infection was possible by subtraction of cDNA from the 4 described pools against cDNA derived from uninoculated leaves and germinated spores. The library was subsequently sub-divided in 4 parts based on sequence tags added during library construction. 20,736 cDNAs were cloned in pGem-T (Promega) for sequencing.

### Sequencing and data processing

The 20,736 cDNA clones were sequenced using an ABI 3730xl DNA sequencer (AME Bioscience) at the catfish genetic research facility (USDA-ASR, Stoneville, MS). The conversion of the electropherogram into base and quality files was performed using Phred [42]. The EST sequences were first cleaned of polyA, polyT, and low complexity sequence using SeqClean from TIGR . Contig assembly was done using the TIGR Gene Indices Clustering Tools (TGICL)  after removing vector and tag sequences. It uses a slightly modified version of NCBI's megablast, and the resulting clusters are then assembled using the CAP3 assembly program (Huang and Madan. 1999). Annotations for the sequences were obtained by Blast against the TIGR plant and fungal sequence databases and Uniprot database. The ESTs were submitted to NCBI Genbank dbEST under the accession numbers FE674093 to FE712011.

### RNA extraction

RNA extraction and cDNA synthesis from leaf tissues of common bean cv Early Gallatin infected with either *U. appendiculatus *race 41 (virulent strain) or race 49 (avirulent strain) and from soybean tissues infected by *P. pachyrhizi*, were performed as described by Libault *et al.*, 2008. Briefly, RNAs were extracted from ground frozen tissues using TRIzol@reagent (Invitrogen, Carlsbad, Calif.) and purified by two phenol/chloroform extractions. The RNAs were treated with TURBO DNA-*free *enzyme (Ambion) to remove all DNA contaminants. cDNA synthesis was prepared from 5 μg of RNA using the MMLV reverse transcriptase (Promega, Madison, WI).

### Quantitative PCR Primer Design

The qRT-PCR primers were designed with primer3 software  using the following criteria, Tm of 60°C, PCR amplicon length from 80 to 125 bp, primer sequence length from 19 to 23 nucleotides with guanine-cytosine contents from 40% to 60% (see additional file 4: excel file of the qRT-PCR primers).

### qRT-PCR reaction conditions and data analysis

The qRT-PCR on bean leaf tissues were performed in a 384-well plate format (7900 HT Sequence detection System; Applied Biosystems, Foster City, CA). The qRT-PCR of soybean leaf, pod, and stem tissues was performed with a 96-well plate qRT-PCR machine (7500 Real-Time PCR System; Applied Biosystems, Foster City, CA). Data analysis was performed as described by Libault *et al. *(2008) with modifications [43]. The data collection was performed during 40 cycles for bean but 45 cycles for soybean with an Rn threshold set at 0.2 for Ct value determination. The ratios of the expression level were transformed into a Log 2 base for clustering in Gene traffic software using a hierarchical clustering algorithm. A t-test was used to assess the statistical differences of the mean of the ratio for each sample at each time point.

The identification of a reference gene for qRT-PCR normalization was made using geNorm software [27]. This software calculates the mean pairwise variation (based on geometrical mean) for each gene and compares these values among these genes. A high mean pairwise variation is found for gene with low expression stability.

## Authors' contributions

ST contributed to the sequences production and analysis, performed the expression analysis and drafted the manuscript; TJ performed the bioinformatic analysis. KBC produced the plant material and contributed to the sequences production; BS sequenced the clones; DX supervised the bioinformatic part of the project and worked over the draft version of the manuscript; BC and HTN supervised the project and worked over the draft version of the manuscript; GS conceived and supervised the project and worked over the draft version of the manuscript. All authors read and approved the final manuscript.

## Supplementary Material

Additional file 1 **List of all the 10,581 unisequences with their associated functional annotation and putative function.** Click here for file

Additional file 2 **List of the 1,704 unisequences having a hit with a BAC-end sequence from the bean BAC library**. Click here for file

Additional file 3 **Ratio of the expression level of the 90 regulated genes at the 6 tested time points.** Click here for file

Additional file 4 **List of the qRT-PCR primers used for the different analysis in common bean.** Click here for file
